# Radical Cure of Hydrocele

**Published:** 1882-04

**Authors:** 


### Abstracts of Hospital Reports.

---

### THE RADICAL CURE OF HYDROCELE.

I desire to bring before the readers of the *British Medical Journal* a procedure, which of late I have practiced with complete success, as a means for effecting the cure of hydrocele. Having tapped the cyst with a short but rather large trocar and canula, when the flow of liquid ceases, I charge a long but slender silver spoon or spatula with a small quantity of finely powdered iodoform, and slip it through the comparatively wide canula into the cavity of the tunica vaginalis, or cyst, as the case may be. A few movements of the spatula will serve to bring the iodoform into contact with different portions of the cyst wall, and, in the event of the tumor having been of large size, a second, or even third, spatulaful of iodoform may be introduced, care being taken to apply some of the powder to the upper and lower extremities of the cyst. After withdrawal of the spatula, the canula is to be removed, when the puncture may be closed by holding the skin pinched between the finger and thumb for a few minutes.

The patient experiences but little pain either during or subsequent to the operation, and, although it is not desirable that the scrotum should be subjected to rough usage, yet it is by no means necessary to keep the patient confined in his room. From my experience of the iodoform treatment, I fully anticipate that all who may give it a trial will find this method to contrast favorably with the sometimes uncertain, often painful, and always disagreeable injection of an iodine solution ; whilst, as for the German plan of laying open the cyst cavity, and drawing it under antiseptic dressings, I consider the measure an unnecessarily severe one, quite uncalled for in the majority of cases.—P. J. HAYES, F.R.C.S., Ed., in *British Medical Journal*.

